# Species Phylogeny versus Gene Trees: A Case Study of an Incongruent Data Matrix Based on *Paphiopedilum* Pfitz. (Orchidaceae)

**DOI:** 10.3390/ijms222111393

**Published:** 2021-10-21

**Authors:** Marcin Górniak, Dariusz L. Szlachetko, Natalia Olędrzyńska, Aleksandra M. Naczk, Agata Mieszkowska, Lidia Boss, Marek S. Ziętara

**Affiliations:** 1 Department of Evolutionary Genetics and Biosystematics, University of Gdańsk, 80-309 Gdańsk, Poland; aleksandra.naczk@ug.edu.pl (A.M.N.); agata.mieszkowska@ug.edu.pl (A.M.); marek.zietara@ug.edu.pl (M.S.Z.); 2 Department of Plant Taxonomy and Nature Conservation, University of Gdańsk, 80-309 Gdańsk, Poland; dariusz.szlachetko@ug.edu.pl (D.L.S.); natalia.oledrzynska@ug.edu.pl (N.O.); 3 Department of Bacterial Molecular Genetics, University of Gdańsk, 80-309 Gdańsk, Poland; lidia.boss@ug.edu.pl

**Keywords:** homoploid hybridization, nuclear genes, concatenation, molecular dating, Orchidaceae, *Paphiopedilum*

## Abstract

The phylogeny of the genus *Paphiopedilum* based on the plastome is consistent with morphological analysis. However, to date, none of the analyzed nuclear markers has confirmed this. Topology incongruence among the trees of different nuclear markers concerns entire sections of the subgenus *Paphiopedilum*. The low-copy nuclear protein-coding gene *PHYC* was obtained for 22 species representing all sections and subgenera of *Paphiopedilum*. The nuclear-based phylogeny is supported by morphological characteristics and plastid data analysis. We assumed that an incongruence in nuclear gene trees is caused by ancestral homoploid hybridization. We present a model for inferring the phylogeny of the species despite the incongruence of the different tree topologies. Our analysis, based on six low-copy nuclear genes, is congruent with plastome phylogeny and has been confirmed by phylogenetic network analysis.

## 1. Introduction

In recent years, nuclear genome data (often obtained using new sequencing methods) have shown discrepancies among phylogenetic trees based on various markers. Tree topology conflicts can be explained either by hybridization or incomplete lineage sorting (ILS) [1]. The latter stems from incomplete random sorting of alleles at many loci, independently, due to short intervals. Maddison [2] proposed minimizing deep coalescences (MDC) as an optimization criterion for inferring the species tree from a set of incongruent gene trees. It was assumed in that paper that ILS was the only reason for the discrepancy between topologies [2]. In order to determine which of the aforementioned processes may be responsible for incongruence in tree topologies, analyses based on the molecular clock were applied [3], in addition to gene coalescence time analysis for a selected group [3,4]. Determining the coalescence time of a particular molecular marker, compared with species divergence time, enables the elimination of ILS as a factor influencing the topology of the phylogenetic tree [3]. However, determining the real coalescence time requires knowledge of the effective population size, ancestor, and generation time factors, which are unknown for many groups. Distinguishing between hybridization and ILS is based on the fact that the latter is a random process that stems from the nature of allele sorting. In contrast, hybridization is not random, and it shortens the genetic distance between species within the introduced markers. Differences between those processes have been used in order to identify them [5]. However, the distinction between these processes requires the analysis of many loci. As the history of many groups is highly reticulate, methods have been developed that include both ILS and hybridization [6,7]. Here, we used multiple nuclear and plastid markers with multispecies coalescent methods and time divergence analysis to understand the processes that gave rise to the lineage of the Asiatic genus *Paphiopedilum*. We also explain the reason for the conflict in the phylogenetic tree topologies constructed on the basis of nuclear markers.

For the purpose of this article, the taxonomy of the genus *Paphiopedilum* proposed by Cribb [8] was used. This Asiatic genus was established by Pfitzer in 1886. It is one of five genera within the subfamily Cypripedioideae (=Cypripediaceae). The intrageneric division of *Paphiopedilum* is based mainly on floral morphology and leaf types [8,9,10,11,12,13]. Plastid DNA analyses [14,15,16] strongly support the infrageneric approach (based on morphological characters) proposed by Cribb [8], which distinguishes three independent evolutionary lineages—namely, three monophyletic clades, corresponding to the subgenera *Parvisepalum*, *Brachypetalum,* and *Paphiopedilum*. Relationships between sections in the subgenus *Paphiopedilum* was also confirmed by analysis of plastid DNA [14,15,16], which revealed the existence of two evolutionary lineages—one including single-flowered species (sections *Barbata* and *Paphiopedilum*) and the other including multi-flowered ones (i.e., sections *Coryopedilum*, *Pardalopetalum*, and *Cochlopetalum*). This was also confirmed by analysis of the plastome [17]. It is worth noting that the currently accepted phylogeny, reconstructed based on plastid data, is supported by morphological data [14], yet it seems not to be supported by nuclear genes researched to date. The *XDH* [15] marker indicates that section *Paphiopedilum* and all three multi-flowered sections are sister groups, which is also true for the *LFY* [16] marker with the exception of section *Cochlopetalum*-*LFY* supports *Cochlopetalum* being a sister group of section *Barbata.* On the other hand, *RAD51* [16] indicates that section *Cochlopetalum* is sister to all other sections of the subgenus *Paphiopedilum*. None of the aforementioned nuclear markers (i.e., the markers *LFY*, *XDH,* and *RAD51*) supports subgenus *Paphiopedilum*, and while the *ACO* and *DEF4* nuclear markers indeed support it strongly [16], they do not resolve relationships (polytomy) within this taxon. It is worth noting that tree topology incongruence of different nuclear markers [15,16] in the subgenus *Paphiopedilum* concerns sections *Barbata* and *Cochlopetalum*.

## 2. Results

### 2.1. Molecular Clock Phylogeny

**Figure 1:**
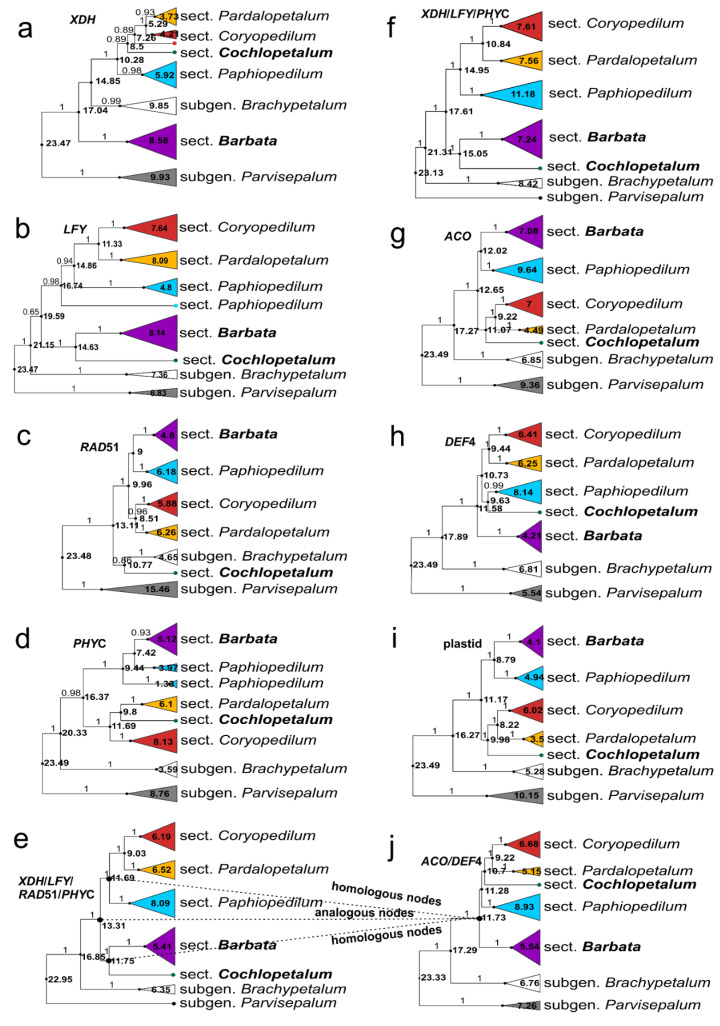
Time-calibrated gene trees of *Paphiopedilum* genus (maximum clade credibility trees) resulting from BEAST analysis of the nuclear data: (**a**)—*XDH*; (**b**)—*LFY*; (**c**)—*RAD51*; (**d**)—*PHYC*; (**e**)—combined *XDH*/*LFY*/*RAD51*/*PHYC*; (**f**)—combined *XDH*/*LFY*/*PHYC*; (**g**)—*ACO*; (**h**)—*DEF4*; (**j**)—*ACO*/*DEF4*; and (**i**)—plastid combined data. Posterior Probability (PP) values > 0.85 are indicated above branches. Numbers at nodes are divergence times based on a strict (all except plastid and *LFY* trees) and relaxed (plastid and *LFY*) clock analysis. The names of taxa with conflicting positions are in bold type. Classification to clades follows Cribb [8].

Bayes Factors between different clock models of data matrices, statistics for one of the most parsimonious trees, and MrModelTest results are shown in Table 1, Table 2 and Table S1, respectively. In all analyses, the species of the subgenus *Parvisepalum* were sister to all the remaining species of the genus (Figure 1 and Figures S1–S4). The *XDH*, *LFY*, and *RAD51* markers (Figure 1a–c, respectively) treated separately do not support subgenus *Paphiopedilum*, whose sections were intermixed with species of subgenus *Brachypetalum*. *PHYC* analysis supported subgenus *Paphiopedilum* only moderately (Posterior Probabilities (PP) = 0.98), (16.4 Ma, 95% Highest Posterior Density (HPD): 11.1-22.1) (Figure 1d). Individual analyses of all the mentioned markers (*XDH*, *LFY*, *RAD51*, *PHYC*) showed incongruence between trees, in relation to entire sections of subgenus *Paphiopedilum* (sections *Barbata* and *Cochlopetalum*). In *XDH* analysis (Figure 1a), multi-flowered species (PP = 0.89), (8.5 Ma, 95% HPD: 5.3–12.9) (sections *Coryopedilum*, *Pardalopetalum*, *Cochlopetalum*) were sister to the nominal one (PP = 1.0), (10.3 Ma, 95% HPD: 6.4–15.1). Section *Barbata* and subgenus *Brachypetalum* were in a polytomy with the above-mentioned sections. In *LFY* tree analysis (Figure 1b), *Coryopedilum* and *Pardalopetalum* were sister to *Paphiopedilum* (PP = 0.98), (16.7 Ma, 95% HPD: 13.3–20.7), and section *Barbata* was sister to section *Cochlopetalum* (PP = 1.0), (14.6 Ma, 95% HPD: 11.6–18.3). In *RAD51* tree analysis (Figure 1c), *Coryopedilum* and *Pardalopetalum* were sister to *Paphiopedilum* and *Barbata* sections (PP = 1.0), (10 Ma, 95% HPD: 7.1–13.2). *Cochlopetalum* was sister to subgenus *Brachypetalum*, although lacks support (PP = 0.86), (10.77 Ma, 95% HPD: 7.41–14.55). In *PHYC* analysis (Figure 1d), *Coryopedilum* with *Pardalopetalum* and *Cochlopetalum* (PP = 1.0), (11.69 Ma, 95% HPD: 7.1–16.78) were sister to *Paphiopedilum* with *Barbata* (PP = 1.0), (9.4 Ma, 95% HPD: 5.0–14.8). A similar topology was obtained in the analysis of the combined plastid data (Figure 1i). Both analyses (*PHYC* and combined plastid) are congruent with morphological analysis [14].

The combined analysis of the four markers (*XDH*/*LFY*/*RAD51*/*PHYC*–Figure 1e) strongly supports subgenus *Paphiopedilum* (PP = 1.0), indicating a common origin of its species and its monophyly. Its tMRCA (time to the Most Recent Common Ancestor) in this analysis equaled 13.3 Ma (95% HPD: 10.0–16.9). On the other hand, exclusion of *RAD51* from the analysis changed tMRCA to 17.6 Ma (95% HPD:13.4–21.86) (Figure 1f). Conversely, the *ACO* (Figure 1g) and *DEF4* (Figure 1h) markers strongly support (PP = 1.0) subgenus *Paphiopedilum*, both when treated separately and in the combined analysis (Figure 1j). Separately, they produced the following tMRCA results: 12.67/11.6 Ma, respectively, (95% HPD: 9.6–16.3/8.4–15.1) (Figure 1g,h), while the combined analysis of these markers indicated 11.7 Ma (95% HPD: 9.1–14.6) (Figure 1j), in accordance with the combined plastid marker analysis (11.2 Ma, 95% HPD: 6.83–16.3) (Figure 1i). A multi-flowered species clade (*Coryopedilum* with *Pardalopetalum* and *Cochlopetalum*) and single-flowered species clade (*Paphiopedilum* and *Barbata*) were not supported by *ACO*, *DEF4,* and *ACO*/*DEF4* analysis (polytomy occurred). Plastid data analysis strongly supported both of the above-mentioned clades (PP = 1.0; 10 Ma, PP = 1.0; 8.8 Ma, respectively) (95% HPD: 6.14–14.8/5.0–13.3, respectively) (Figure 1j).

### 2.2. Molecular Phylogenetics and Network Analysis

**Figure 2:**
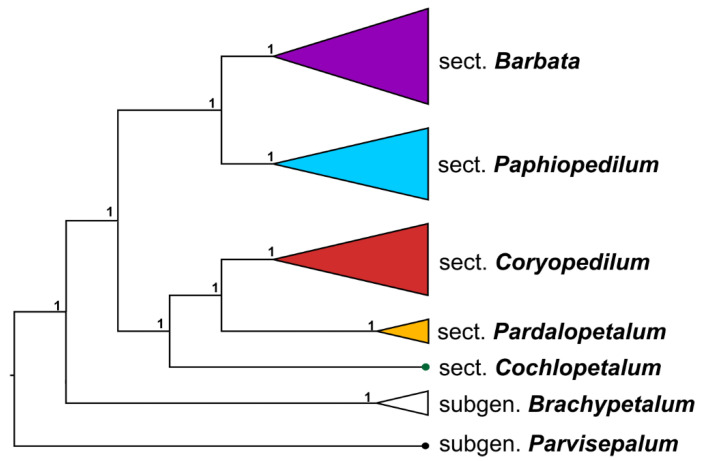
Nuclear gene tree of *Paphiopedilum* genus based on combined analysis of *ACO/DEF4/PHYC/XDH*_1/*XDH*_2/*LFY*_1/*LFY*_2/*RAD51_*1/*RAD51_*2 (see Table 3). PP values are indicated above branches.

**Figure 3:**
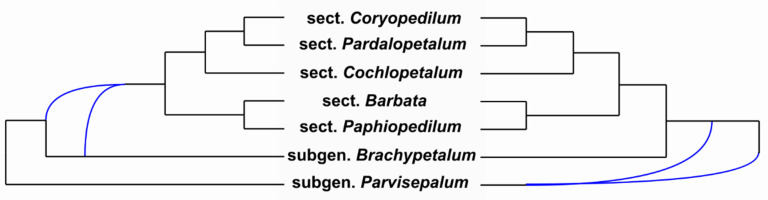
Two of the most probable Bayesian MCMC phylogenetic networks (log = −13.19688, log = −13.75276) using gene tree topologies (nuclear—*ACO*, *DEF4*, *PHYC*, *XDH*, *LFY*, *RAD51*, and plastid tree). The Blue line indicates possible scenarios of hybridization.

Concatenated analysis of all nuclear markers with modification of data matrices (as shown in Table 3) indicated that (1) subgenus *Paphiopedilum* was monophyletic and (2) single-flowered species (sections *Barbata* and *Paphiopedilum*) and multi-flowered species (i.e., sections *Coryopedilum*, *Pardalopetalum*, and *Cochlopetalum*) were monophyletic. All the internal nodes of the tree were supported by PP = 1.0 (Figure 2). Network analysis using Bayesian MCMC_GT showed the same topology of the tree listed in a 95% credible set of topologies (percent = 53.94). In addition, eleven reticulation scenarios were detected. Two of the most probable networks (log = −13.19688, log = −13.75276) are depicted in Figure 3.

## 3. Discussion

### 3.1. Molecular Dating

Hitherto, none of the four low copy nuclear markers analyzed by Górniak et al. [15] and Guo et al. [16] showed a topology fully congruent with plastid and morphological analysis. Our results, based on the *PHYC* analysis, support the morphological data and confirm the existence of two evolutionary lineages within subgenus *Paphiopedilum*, the first of which includes the sections *Barbata* and *Paphiopedilum*, while the second one includes the multi-flowered species (i.e., sections *Coryopedilum*, *Pardalopetalum*, and *Cochlopetalum*).

**Figure 4:**
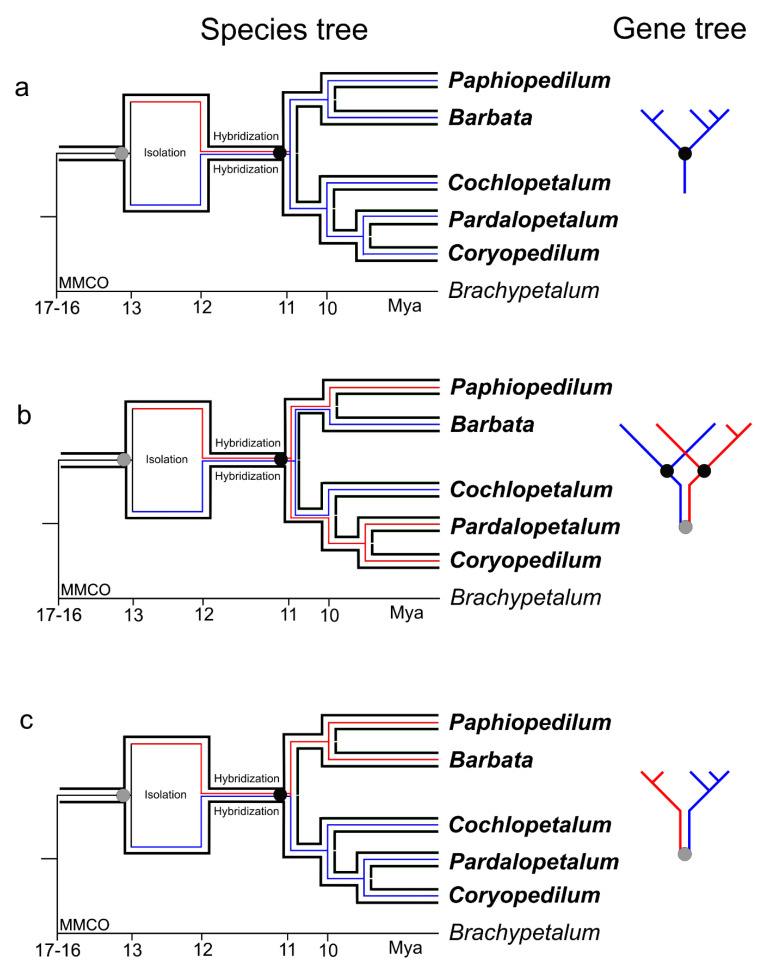
The species tree (black line) on which plastid gene tree (**a**), nuclear gene trees *LFY* (**b**), and *PHYC* (**c**) were applied. The illustration explains the reason for conflicts between gene trees and species trees within the subgenus *Paphiopedilum*. MMCO is the Mid-Miocene Climatic Optimum. Blue and red lines represent different gene copies (alleles/haplotypes) that diverged as a result of isolation/speciation. For the plastid gene tree, it was assumed that, after the hybridization process, only one haplotype remained (**a**). The nuclear gene tree for *PHYC* (**c**) and the plastid gene tree (**a**) have identical topology but different nodes with respect to their MRCA—a proto*-Paphiopedilum* subgenus (gray node) and *Paphiopedilum* subgenus (black node), respectively. The gene trees for *LFY* (**b**) and for *PHYC* (**c**) have different topologies but the same node with respect to their MRCA (gray node).

Although gene duplication and ILS cannot be rejected as the reason for phylogenetic tree topology incongruence among the *XDH*, *LFY*, *RAD51,* and *PHYC* genes (Figure 1a–d), where the subgenus *Paphiopedilum* is concerned, ancestral hybridization is a more probable explanation, as well as a parsimonious one. The proof for this hypothesis lies in the phylogenetic trees based on the molecular clock and phylogenetic network analysis. Two groups of trees can be distinguished on the basis of the obtained results. The first group (*ACO*, *DEF4*, plastid DNA trees; Figure 1g–i) strongly supports (PP 1.0) the subgenus *Paphiopedilum* in its current circumscription and shows a strong division (only the plastid DNA analysis) into two groups corresponding to morphology. The tMRCA for subgenus *Paphiopedilum*, both for the plastid DNA and combined nuclear (*ACO*/*DEF*) analyses equals 11.17 Ma (95% HPD: 6.8–16.3) and 11.7 Ma (95% HPD: 9.1–14.6), respectively (Figure 1i,j). The divergence times of specific sections within the subgenus are also highly similar. The second group of trees (*LFY*, *RAD51*, *XDH*, *PHYC*) (Figure 1d) does not support the monophyly of the subgenus on its own; however, the combined analysis shows a highly supported tMRCA of the subgenus *Paphiopedilum* of ca. 13.31 Ma (95% HPD: 10.0–16.9) (Figure 1e). Although the value is only 2 million years higher, compared with the combined plastid and *ACO*/*DEF4* genes analysis, it increases to 6 My when *RAD51* is excluded from the analysis (Figure 1f). The nodes on the phylogenetic trees obtained from the analyses of the combined *XDH*/*LFY/RAD51/PHYC* (Figure 1e) and *ACO*/*DEF4* (Figure 1j) matrices referring to MRCA of the subgenus *Paphiopedilum* are “analogous” in the case of both analyses (Figure 1e,j). The difference within the time scale of these nodes stems from the analysis of one (plastid genome, *ACO*, *DEF4*) (Figure 4a) or both parental alleles (*XDH*, *LFY, RAD51, PHYC*) (Figure 4b,c). On the other hand, the nodes resulting from the same speciation event (the “homologous nodes”) refer to different terminal taxa (Figure 1e,j). We believe that the tMRCA of the *Paphiopedilum* subgenus, calculated on the basis of the *XDH*/*LFY/RAD51/PHYC* matrix, does not refer to the ancestor of the subgenus *Paphiopedilum* but to a proto-*Paphiopedilum* ancestor (subgenus *Paphiopedilum*) from before the hybridization event. It seems that ancestral hybridization that gave rise to a new evolutionary lineage (hybrid origin) that subsequently diversified may be interpreted as ILS.

### 3.2. Geological: Climate Changes through Miocene

According to Hall [18], between 60 and 5 million years ago (Neogene era) the Sunda Shelf area was above sea level, thus making migration of plants from continental Asia to Borneo theoretically possible. In such a scenario, climate may have served as a migration limiting factor [19]. The early and middle Miocene epoch was wet and warm, with rainforest across Sundaland [20]. Southward migration from continental Asia to Sundaland during the Miocene period has been documented for many plant species, e.g., *Lithocarpus* (Fagaceae) [21], *Pseuduvaria* (Annonaceae) [22], *Begonia* (Begoniaceae) [23], and *Alocasia* (Araceae) [24]. A decline in the sea level enabled a periodic exchange of organisms between regions that were otherwise isolated. Based on geological reconstructions and palynological analyses, de Bruyn et al. [25] concluded that Miocene flora and fauna were divided between two regions: the northern (Indochina) and the southern (Borneo) parts of the Thai-Malay Peninsula, within the mid-Miocene climatic optimum (MMCO), approximately 17-15 Ma [26] or 16-14 Ma [27]. Interestingly, this period correlates closely with the *Brachypetalum*/proto-*Paphiopedilum* subgenus divergence time of 16-17 Ma (Figure 1e,h,i,j). Diversification of proto-*Paphiopedilum* subgenus into two subpopulations and species formation could have stemmed from geographic isolation generated by a sudden water level rise, which occurred approximately 13.8 Ma (the Serravallian Stage) [20]. Hybridization of both lineages (species/divergent populations) had to occur more than 11 million years ago. Afterward, rapid radiation of the subgenus *Paphiopedilum* could have caused differentiation into five sections. Each of the sections is highly diversified morphologically [8], and it seems highly probable that they also arose in geographical isolation within the period of strong ocean level fluctuations in the mid-Miocene, about 10-11 Ma (Figure 1). This time agrees with several significant sea-level fluctuations that occurred in the Miocene epoch. Sea levels declined during the Serravallian Stage (at 13.8 Ma, 12.8 Ma, 11 Ma), and during the late Miocene (the earliest Tortonian), it was at −90 m [20]. Guo et al. [16] and Tsai et al. [28] proved that sea-level fluctuations had an impact on the evolution of *Paphiopedilum*. Both authors [16,28] indicate that in situ speciation within islands accelerated species divergence in the subgenus *Paphiopedilum*. This rapid radiation can also be supported by the unresolved tree topologies (polytomy) of the markers that originate from one parental lineage (*ACO*, *DEF4,* and individual plastid markers). However, we considered, taking into account our analysis, that the shift of speciation rate in the subgenus *Paphiopedilum* was preceded by the hybridization process. There are, however, many indications in favor of hybridization being a factor contributing to nuclear tree topology inconsistencies with regard to the subgenus *Paphiopedilum.* These symptoms are observed on many levels, such as species diversification, genome rearrangement, and geographic distribution. The subgenus *Paphiopedilum* comprises about 60 species, compared with 4 and 5 species for 2 other subgenera, *Brachypetalum* and *Parvisepalum*, respectively [8]. On the cytological level, the subgenus *Paphiopedilum* is also characterized by multiple duplications regarding 5S rDNA [29]. The geographic range of subgenera *Parvisepalum* and *Brachypetalum* is limited to southeast continental Asia. Subgenus *Paphiopedilum*, with the exception of the section *Paphiopedilum*, also occupies the islands of the Malaysian Archipelago, New Guinea, and the Solomon Islands.

### 3.3. Post-Hybridization Scenario

Processes stemming directly from hybridization might be the cause of the loss of alleles. Müntzing [30] postulated that sorting of chromosomal rearrangements in later-generation hybrids could, by chance, lead to the formation of new populations that were homozygous for a unique combination of chromosomal sterility factors and were partially reproductively isolated from both parental species. Stebbins [31] and Grant [32] proposed a model of homoploid hybrid speciation where two parental species are distinguished by two or more separable chromosomal rearrangements. This leads through a partially sterile hybrid stemming from segregation and recombination to new homozygous recombinant types [33]. This means that even a whole chromosome of one of the two parents could very quickly be lost in a hybrid population. Folk et al. [34] show that after the crossing-over, fertile hybrids in generation F_2_ will be heterozygotes carrying recombined chromosomes. After several generations, sorting or coalescence of all parental alleles may occur. The effect will be a mosaic of parental genomes. Folk et al. [34] call the divided fragments of chromosomes of the same evolutionary history “h-genes” (from the hybrid event). If the aforementioned processes had occurred before the differentiation of two evolutionary lineages (single- and multi-flowered), trees of the nuclear genes should correspond to the species tree since they will not show the ancestral hybridization due to carrying the same ancestral copy inherited from only one of the parents. In other words, differentiation and genetic distance should be consistent with the speciation processes that occurred within the subgenus *Paphiopedilum*. Such a process is shown in Figure 4a. In our analyses, this applied to plastid and nuclear *ACO* and *DEF4* genes. Lack of topology/divergence time conflict among the plastid DNA suggests unidirectional hybridization, which is common in the family Orchidaceae [35]. If both parental alleles had survived in the gene pool after the hybridization event and were introduced to the populations of the multi-flowered section ancestor, as well as the *Barbata* and *Paphiopedilum* sections ancestor, they could have entered different sections randomly during divergence (Figure 4b). This process would have resulted in phylogenetic gene trees being incongruent with the species tree obtained on the basis of nuclear DNA analysis, which would support different tree topologies referring to various sections of the subgenus *Paphiopedilum.* In this case, only sections *Barbata* and *Cochlopetalum* would be in conflict. It is worth noting that both of these sections carry a higher than the basic (plesiomorphic) number of chromosomes. The question remains whether the decomposition of chromosomes observed independently in representatives of sections *Barbata* and *Cochlopetalum* stems from inheriting a particular set of genes/chromosomes after hybridization that had to be decomposed due to their incompatibility. The third possible scenario is represented by the *PHYC* gene analysis. The *PHYC* gene, in turn, corresponds with the analysis of the plastid DNA/morphology; however, we also believe it to have resulted from a random distribution of two parental alleles to each of the populations, but in accordance with speciation processes that occurred after the hybridization process (Figure 4c).

### 3.4. Reconstruction of the Phylogeny

Conducting a combined phylogenetic analysis with conflicted markers is a difficult challenge. Usually, the incongruent nodes are removed before the combined analysis is carried out. Unfortunately, this eliminates the possibility to determine the phylogenetic relationships of the conflicted taxa. In the case of a conflict between two sets of markers (e.g., cpDNA vs. nrDNA), in order to show the hybridization, a taxon is duplicated, with plastid-only accession remaining in one set (nrDNA characters coded as missing) and nrDNA-only accession in the other (plastid characters coded as missing) [36]. As a result, a phylogenetic location obtained from various markers is shown on one tree [36]. In our analysis, two other approaches were implemented in order to determine the phylogenetic relationships within the genus *Paphiopedilum*. The first one was based on phylogenetic network analysis using Bayesian MCMC from gene tree topologies. The second one comprised a combined analysis of the nuclear markers according to the presented scheme in spite of their topology conflicts (Table 3). The results of both analyses are identical and consistent with the morphological hypothesis and the plastid DNA analysis. Moreover, the network analysis confirmed the assumption of ancestral hybridization being the reason for the conflict of various marker topologies (Figure 3). A precise description of the subgenus phylogeny is complicated by the mosaic genotype setup of the ancestral populations of each of the sections, divided randomly among them. Only a process of revealing a large portion of the whole genomes would make it possible to finally determine the relationships. In our work, we indicated how these relationships will appear as.

## 4. Materials and Methods

### 4.1. DNA Isolation, Amplification, and Sequencing

DNA from representatives of Cypripedioideae was extracted from 27 species (Supplementary Table S2). Genomic DNA was extracted from 20 mg of specimens (parts of leaves) dried in silica-gel [37] using a DNA Mini Plant Kit (A&A Biotechnology, Poland). Descriptions of laboratory procedures such as primers used, specific conditions for polymerase chain reaction (PCR), purification, and sequencing for low-copy *XDH* and plastid *matK* genes were given in a separate paper [15]. Low-copy nuclear gene *PHYC* was amplified using PHYC_F (5′GTTYCATGARGATGAGCATGG3′) and PHYC_R (5′GCTCCTCCCCAYTTGATTTC3′) primers designed specifically for this study. The PCR reaction was performed using Dream Taq PCR Master Mix (2x) (Thermo Scientific). In cases where heterogeneity of nucleotides was observed, an additional step of DNA cloning was conducted using the pJET 1.2 vector (Thermo Scientific CloneJET PCR Cloning Kit). Competent cells were obtained using *Escherichia coli* MC1061 [38] and calcium chloride. PCR amplification was performed using pJET1.2_F and pJET1.2_R primers, included in the kits. The High-Pure PCR Product Purification Kit (Roche Diagnostic GmbH, Germany) was used for the purification of PCR products, in accordance with the protocol specified by the manufacturer. The sequencing procedure was performed using the Big Dye Terminator v3.1 Cycle Sequencing Kit (Applied Biosystems, ABI, Warrington, Cheshire, UK), with the same primers as for the amplification step. GenBank accession numbers are presented in Supplementary Table S2.

### 4.2. Molecular Phylogenetic and Time Divergence Analysis

All sequences, except for *PHYC* and several of *matK* (11 sequences) and *XDH* (10 sequences), were downloaded from GenBank (Supplementary Table S2). To estimate the tMRCA for *Paphiopedilum*, representatives of all Cypripedioideae genera (*Paphiopedilum*, *Phragmipedium*, *Mexipedium*, *Cypripedium,* and *Selenipedium*) were used in the analysis. The Bayesian strict molecular clock approach implemented in BEAST v. 1.8.1 [39] was used for *matK*, *PHYC,* and *XDH* matrices and combined analysis. The age for the root of the tree (Cypripedioideae) was set to a normal prior distribution with a mean of 64 Ma and a standard deviation of 4.0 to the resulting age estimated for Cypripedioideae based on the analysis of *matK*+*rbcL* [40]. The original calibration point source was based on Ramirez et al. [41]. Trees are provided in Supplementary Figures S1–S4. Based on this analysis, the prior age parameter for the tMRCA for *Paphiopedilum* clade (the root of the tree) was set to 24 Ma with normal distribution and a standard deviation of 2.0. Six different low-copy nuclear genes (*ACO*, *DEF4*, *XDH*, *LFY*, *RAD51*, and *PHYC*) and a combined plastid matrix (*ycf1*, *atpI-atpH*, *trnL-trnF, atpF-atpH*, *rpoC2*, *accD*, *rbcL*, and *matK*) were analyzed. In Addition, two combined nuclear data sets were created based on topologies of previously published trees [15,16] and those of this study (*PHYC*), which were used in the analysis. These are as follows: the monophyly matrix markers *ACO*/*DEF4* (which strongly support subgenus *Paphiopedilum,* but do not resolve relationships within the subgenus–polytomy that occurred); and the non-monophyly matrix markers *XDH*/*LFY/RAD51/PHYC* (subgenus *Paphiopedilum* is not supported by PP and topologies of the trees based on these markers are incongruent with each other). The aim of the latter analysis was to check whether the concatenated matrix of the above markers also supports the monophyletic character of the subgenus *Paphiopedilum*. Sequence alignments were obtained using Mafft v. 7 [42] and then analyzed by SeaView v. 4.7 [43]. The Bayesian strict molecular clock (for nuclear data) and the uncorrelated relaxed molecular clock (for combined plastid data) approaches implemented in BEAST v. 1.8.1 [39] were used. For all data matrices, the birth–death process [44] was chosen as the speciation process. Two runs were performed in BEAST, with 20 million generations each and a sampling frequency of 1000. The MrModeltest v. 2 [45] was used in order to choose the best-fitting evolutionary model by the Akaike Information Criterion. Tracer v. 1.6 [46] was used to analyze Log files to assess the convergence. The combined effective sample sizes for all the chosen parameters were larger than 200. All the obtained trees were combined with LogCombiner v. 1.8.1 [39], with a burn-in of 25%. Finally, the maximum credibility tree was produced using TreeAnnotator v. 1.8.1 [39]. The molecular clock was chosen based on the Bayes factor (2log_e_(BF)) using the marginal likelihoods of the model estimated using the stepping-stone/path-sampling methods in BEAST v. 1.8.1 [39]. Evidence against the null model, which is the one with lower marginal likelihood, was estimated based on Kass and Raftery [47]. For the analysis, 2 > 2log_e_(BF) > 6 indicates positive evidence against the null model; 6 > 2log_e_(BF) > 10 indicates strong evidence against the null model; 2log_e_(BF) > 10 indicates very strong evidence against the null model.

### 4.3. Phylogenetic Network Analysis

The small data matrix (one species from each section) was created and analysed using MrBayes v. 3.2.5 [48]. The posterior probabilities of clades were estimated by sampling trees from the PP distribution using Markov chain Monte Carlo (MCMC) simulations. Six chains were run for 10,000,000 generations. Trees were sampled every 100 generations. The first 25,000 trees were discarded as the burn-in samples. The remaining trees were used to assess topology, PPs, and gene tree distributions. The internal nodes with PP lower than 0.97 in gene trees analysis were collapsed: [*XDH*]—((((A,B),(C),E),(D),(F)),G); [*RAD51*]—(((F,C),((A),(B),(E),(D))),G); [*PHYC*]—((F,(((B),(A),(C)),(E,D))),G); [*ACO*]—((F,(((A,B),C),(E),(D))),G); [*DEF4*]—((F,((A,B),(C),(E),(D))),G); [*LFY*]—((F,(A,B),(E),(C,D)),G); and [Plastid] ((F,(((A,B),C),(E,D))),G). These were used as input trees in *MCMC_GT* analysis in PhyloNet software v. 3.8.0 [49,50]. Nodes presented in Newick format were as follows: A—sect. *Coryopedilum*; B—sect. *Pardalopetalum*; C—sect. *Cochlopetalum*; D—sect. *Barbata*; E—sect. *Paphiopedilum*; F—subgen. *Brachypetalum*; and G—subgen. *Parvisepalum*. *MCMC_GT* infers phylogenetic networks using Bayesian MCMC from gene tree topologies using a multispecies network coalescent framework (MSNC) [51]. MSNC incorporates hybridization in addition to incomplete lineage sorting (ILS). The length of the MCMC chain was 2,000,000 with 200,000 of iterations in burn-in period. The sample frequency was 1000. The maximum number of reticulation nodes in the sampled phylogenetic networks was the default value—infinity. Network/Trees generated by PhyloNet were visualized by Dendroscope v. 3.6.3 [52].

### 4.4. Using Nuclear Genes for Inferring Phylogeny

All six nuclear genes were concatenated into one matrix. Data matrices for *ACO*, *DEF4* (which strongly support subgenus *Paphiopedilum* but do not resolve relationships within the subgenus), and *PHYC* (which supports *Paphiopedilum* subgenus only moderately and resolves the relationships within the subgenus-congruent with plastid phylogeny) were used without any modifications. Data matrices for *XDH*, *LFY*, *RAD51* (topology incongruence concerns the entire sections—*Barbata* and *Cochlopetalum*) were duplicated, and some taxa (representing entire sections) were deleted, as shown in Table 3. The taxa removed from one submatrix were used in the other one, and vice versa. For example, in the case of the *LFY* matrix sections, *Barbata* and *Cochlopetalum* are sister groups. This relationship is inconsistent with both morphological and plastid DNA analyses. In such a case, the representatives of both sections were removed from one submatrix, while all of the remaining sections’ representatives (*Pardalopetalum*, *Coryopedilum,* and *Paphiopedilum*) were removed from the other copy. As a result, all of the data (sequences) were used in the analysis. *Brachypetalum* and *Parvisepalum* subgenera representatives were included in both matrices. During the analyses, we assumed that the topology conflicts stem from ancestral hybridization/ILS/genome duplication, and a specific cause is unimportant. The goal of the analyses was to obtain the species phylogeny in spite of the topology incongruence of the particular phylogenetic gene trees. Therefore, a matrix modified in this way (see Table 3) was subjected to Bayesian inference in MrBayes v. 3.2.5 [48] for inferring the phylogeny of the genus *Paphiopedilum*. The analysis was performed as described in Section 4.3.

## Figures and Tables

**Table 1:** Bayes factors between different clock models of data matrices.

Data Matrix	Model Comparison	Log Marginal Likelihood	Bayes Factor (2 BF)	Evidence against H_0_
*ACO*	Relaxed/B and D # vs. Strict/B and D *	−4499.32 vs. −4496.78	5.08	Weak
*DEF4*	Relaxed/B and D # vs. Strict/B and D *	−3701.28 vs. −3700.92	0.72	Inconclusive
*PHYC*	Relaxed/B and D # vs. Strict/B and D *	−1665.58 vs. −1662.93	5.3	Weak
*XDH*	Relaxed/B and D # vs. Strict/B and D *	−2143.99 vs. −2143.49	1.0	Inconclusive
*LFY*	Strict/B and D # vs. Relaxed/B and D *	−12,001.96 vs. −11,972.86	58.2	Very strong
*RAD51*	Relaxed/B and D # vs. Strict/B and D *	−2908.64 vs. −2906.61	4.06	Weak
plastid	Strict/B and D # vs. Relaxed/B and D *	−15,868.13 vs. −15,848.6	39.06	Very strong

B and D —the birth–death process; #—H_0_, the null hypothesis (with lower marginal likelihood); *—selected model.

**Table 2:** Statistics of one of the parsimonious trees.

Matrix	*ACO*	*DEF4*	*ACO/DEF4*	*PHYC*	*XDH*	*LFY*	*RAD51*	*XDH/LFY/ RAD51/ PHYC*	Plastid	Nuclear Combined Referring to Table 1
No. of taxa	22	23	21	23	23	21	23	20	23	19
Included positions in matrix	1393	1241	2634	799	909	3368	892	5948	8731	13,117
Variable site	296	246	518	55	101	862	193	1154	442	2093
Parsimony-uninformative sites	163	128	281	21	63	377	113	581	182	1270
Parsimony-informative sites	133	118	237	34	38	485	80	573	260	823
Consistency index (CI)	0.9	0.95	0.91	0.9	0.95	0.8	0.88	0.8	0.88	0.9
Retention index (RI)	0.88	0.95	0.89	0.94	0.96	0.79	0.86	0.79	0.91	0.82

**Table 3:** Combination of data partitions (nuclear-combined data matrix) and taxa used for phylogenetic analyses. Shaded boxes indicate sequences, and white boxes indicate excluded data (“missing data”) in the matrix.

Taxa/Markers	*ACO*	*DEF4*	*PHYC*	*XDH_*1	*XDH*_2	*LFY*_1	*LFY*_2	*RAD51*_1	*RAD51*_2
*Coryopedilum*									
*Pardalopetalum*									
*Cochlopetalum*									
*Paphiopedilum*									
*Barbata*									
*Brachypetalum*									
*Parvisepalum*									

## Data Availability

Data matrices are available online at https://doi.org/10.6084/m9.figshare.16577810 (last accessed on 22 October 2021).

## Supplementary Materials

The following are available online at https://www.mdpi.com/article/10.3390/ijms222111393/s1.
