# Supplementary material for: Species Phylogeny versus Gene Trees: A Case Study of an Incongruent Data Matrix Based on Paphiopedilum Pfitz. (Orchidaceae)
Source: Int J Mol Sci. 2021 Oct 21;22(21):11393. doi: 10.3390/ijms222111393 (PMC8583834; doi:10.3390/ijms222111393)
Supplement: Supplementary file 1 [file ijms-22-11393-s001.zip › ijms-1400637-supplementary.pdf]

Article

SPECIES PHYLOGENY VERSUS GENE TREES: A CASE STUDY OF AN INCONGRUENT DATA MATRIX BASED ON *PAPHIOPEDILUM* PFITZ. (ORCHIDACEAE)

Marcin Górniak [1*], Dariusz L. Szlachetko[2], Natalia Olędrzyńska[2], Aleksandra M. Naczk[1], Agata Mieszkowska[1], Lidia Boss[3] and Marek S. Ziętara [1]

[1] Department of Evolutionary Genetics and Biosystematics, University of Gdansk, 80-309 Gdańsk, Poland; marcin.gorniak@ug.edu.pl (M.G.); aleksandra.naczk@ug.edu.pl (A.N.); agata.mieszkowska@ug.edu.pl (A.M.); marek.zietara@ug.edu.pl (M.Z.)

[2] Department of Plant Taxonomy and Nature Conservation, University of Gdańsk, 80-309 Gdańsk, Poland; dariusz.szlachetko@ug.edu.pl (D.S), natalia.oledrzynska@ug.edu.pl

[3] Department of Bacterial Molecular Genetics, University of Gdańsk, 80-309 Gdańsk, Poland; lidia.boss@ug.edu.pl

* Correspondence: marcin.gorniak@ug.edu.pl

Supplementary Data

Table S1 MrModelTest results

Data matrix	model selected by MrModelTest	Data matrix	model selected by MrModelTest
ACO	SYM+G	*matK*	GTR+G
DEF4	HKY+I	*rbcL*	F81
XDH	HKY+G	*ycf1*	GTR+I+G
LFY	GTR+I+G	*atpI-atpH*	GTR+I
RAD51	GTR+I	*trnS-trnfM*	HKY
PHYC	HKY+G	*atpF-atpH*	GTR+I
*XDH*_2	HKY+G	*rpoC2*	GTR+I
*LFY*_2	GTR+I	*accD*	GTR+I
*RAD51*_2	GTR+I		

Table S2. Sources of materials and GenBank accession numbers of taxa used in this study

	Taxa	Sources/Vouchers	*ycf1*	*atpI-atpH*	*trnS-trnfM*	*atpF-atpH*	*rpoC2*	*accD*	*rbcL*	*ACO*	*DEF4*	*LFY*	*RAD51*	Sources/Vouchers	*PhyC*	*Xdh*	*matK*
**Subg. *Parvisepalum***	*P. armeniacum* S.C. Chen & F.Y. Liu	NOCC, 2471	KP311695	KP312208	KP312321	KP312560	KP311896	KP312095	KP311796	KP312432	KP312673	-	KP312898	08/07/P	KX886238*	JQ660950	JQ660906
	P. delenatii Guillaumin	NOCC, 1951	JQ182265	KP312209	KP312322	KP312561	JQ182229	KP312096	JQ182211	JQ182154	KP312674	JQ182187	KP312900	29/06/S	KX886239*	JQ660949	JQ660905
	P. hangianum Perner & O.Gruss (Parvisepalum 1)	NOCC, 2501	KP311699	-	-	-	-	-	-	KP312437	-	-	-		-	-	-
	P. micranthum Tang & F.T.Wang (Parvisepalum 1)	NOCC, 1919	KP311698	KP312212	KP312325	KP312564	KP311899	KP312099	KP311799	-	KP312677	-	KP312903	70/06/P	KX886237*	GU004514	KX886268*
	P. vietnamense O.Gruss & Perner (Parvisepalum 1)	NOCC, 2090		-	-	-	-	-	-	-	-	JQ182189	-		-	-	-
**Subg. *Brachypetalum***	*P. bellatulum* (Rchb.f.) Stein	NOCC	JN181516	KP312217	KP312330	KP312569	JN181499	KP312104	JN181465	JN181401	KP312681	JN181424	KP312910	13/07/P	KX886234*	KX886255*	KX886267*
	P. concolor (Lindl. ex Bateman) Pfitzer in H.G.A.Engler & K.A.E.Prantl (eds.)	NOCC, 2511	KP311704	KP312220	KP312333	KP312572	KP311905	KP312107	KP311805	KP312441	KP312684	KP312788	KP312915	25/07/C	KX886236*	JQ660946	JQ660902
	P. godefroyae (God.-Leb.) Stein (Brachypetalum 1)	NOCC, 4626	-	-	-	-	-	-	-	-	-	KP312794	-		-	-	-
	P. niveum (Rchb.f.) Stein (Brachypetalum 1)	NOCC, 6328	KP311710	KP312226	KP312339	KP312578	KP311911	KP312113	KP311811	KP312453	KP312692	-	KP312922	78/08/P	KX886235*	JQ660947	JQ660903
**Subg. *Paphiopedilum***																	
**Sect. *Coryopedilum***	*P. glanduliferum* (Blume) Stein (*Coryopedilum 1*)	NOCC, 6182	KP311713	KP312230	KP312343	KP312582	KP311914	KP312117	KP311814	KP312457	KP312697	KP312803	KP312927		-	-	-
	P. kolopakingii Fowlie	NOCC, 6321	KP311714	KP312231	KP312344	KP312583	KP311915	KP312118	KP311815	KP312458	KP312699	KP312804	KP312929	62/06/P (c.96)	KX886226*	KX886253*	KX886265*
	P. ooii (*Coryopedilum 1*)	-		-	-	-	-	-	-	-	-	-	-	79/05/P (c.2)	KX886225*	KX886252*	KX886264*
	P. philippinense (Rchb.f.) Stein (*Coryopedilum* 2)	NOCC, 2035	KP311715	KP312232	KP312345	KP312584	KP311916	KP312119	KP311816	KP312459	KP312700	KP312805	KP312930		-	-	-
	P. randsii (*Coryopedilum 2*)	-								-	-	-	-	89/06/P (c.62)	KX886232*	KX886254*	KX886266*
	P. rothschildianum (Rchb.f.) Stein	NOCC, 6071	KP311718	KP312235	KP312348	KP312587	KP311919	KP312122	KP311819	KP312463	KP312703	KP312813	KP312933	93/06 (c.2)	KX886227*	JQ660935	JQ660888
**Sect. *Pardalopetalum***	*P. haynaldianum* (Rchb.f.) Stein	NOCC, 2014	KP311724	KP312242	KP312355	KP312594	KP311925	KP312129	KP311825	KP312469	KP312709	KP312821	KP312940	49/06	KX886224*	KX886251*	KX886263*
	P. lowii (Lindl.) Stein	NOCC, 4624	KP311726	KP312244	KP312357	KP312596	KP311927	KP312131	KP311827	-	KP312711	KP312825	KP312942	66/08/P (c.52)	KX886222*	JQ660936	JQ660890
	P. parishii (Rchb.f.) Stein	NOCC, 2628	KP311727	KP312245	KP312358	KP312597	KP311928	KP312132	KP311828	KP312472	KP312712	KP312820	KP312944	82/06/P	KX886223*	JQ660937	JQ660891
**Sect. *Cochlopetalum***	*P. chamberlainianum* (*Cochlopetalum 1*)	-		-	-	-	-	-	-	-	-	-	-	20/08/P	KX886221*	JQ660938	JQ660892
	P. victoria-regina (Sander) M.W.Wood (*Cochlopetalum 1*)	MBG, 921150-1	KP311733	KP312252	KP312365	KP312604	KP311934	KP312139	KP311834	KP312480	KP312721	KP312833	KP312953		-	-	-
**Sect. *Paphiopedilum***	*P. druryi* (Bedd.) Stein 1	MBG, 913218-1	KP311739	KP312258	KP312371	KP312610	KP311940	KP312145	KP311840	KP312488	KP312727	KP312792	KP312963	32/06/K	KX886228*	JQ660939	JQ660894
	P. gratrixianum Rolfe 1	NOCC, 3078	KP311743	KP312262	KP312375	KP312614	KP311944	KP312149	KP311844	KP312492	KP312730	KP312843	KP312966	44/03/K	KX886229*	JQ660944	JQ660900
	P. tranlienianum O.Gruss & Perner	NOCC	KP311757	KP312277	KP312389	KP312629	KP311957	KP312164	KP311858	KP312514	KP312746	KP312852	KP312985	112/07/P	KX886233*	KX886250*	KX886262*
	P. villosum (Lindl.) Stein	MBG, 913229-1	KP311758	KP312278	KP312390	KP312630	KP311958	KP312165	KP311859	KP312515	KP312748	KP312853	KP312987	119/99	KX886216*	JQ660943	JQ660899
**Sect. *Barbata***	*P. barbatum* (Lindl.) Pfitzer	NOCC, 6320	KP311761	KP312281	KP312393	KP312633	KP311960	KP312168	KP311862	KP312519	KP312750	KP312856	KP312990	11/08/P	KX886215*	JQ660940	JQ660895
	P. callosum (Rchb.f.) Stein	NOCC, 25191	KP311763	KP312283	KP312395	KP312635	KP311962	KP312170	KP311864	KP312523	KP312752	KP312859	KP312993	17/06/P	KX886217*	KX886246*	KX886258*
	P. dayanum (Lindl.) Stein	NOCC, 4629	KP311766	KP312286	KP312398	KP312638	KP311965	KP312173	KP311867	KP312526	KP312755	KP312863	KP312996	28/06/P	KX886219*	KX886245*	KX886257*
	P. virens var. javanicum (Reinw. ex Lindl.) Pfitzer (Barbata 1)	-	-	-	-	-	-	-	-	-	-	-	-	121/05/P	KX886214*	KX886244*	KX886256*
	P. javanicum (Reinw. ex Lindl.) Pfitzer (Barbata 1)	NOCC, 6318	KP311769	KP312289	KP312400	KP312641	KP311967	KP312176	KP311869	KP312528	KP312756	KP312865	KP312998		-	-	-
	P. purpuratum (Lindl.) Stein	NOCC, 1615	KP311773	KP312293	KP312404	KP312645	KP311971	KP312180	KP311873	KP312533	KP312760	KP312870	KP313002	88/06/P	KX886218*	KX886247*	KX886259*
**Outgroup**	*Phragmipedium besseae*	-	-	-	-	-	-	-	-	-	-	-	-	136/08/P	KX886240*	JQ660951	JQ660908
	Cypripedium dickinsonianium	-	-	-	-	-	-	-	-	-	-	-	-	Fred Muller s.n.	KX886241*	JQ660952	JQ660909
	Mexipedium xerophyticum	-	-	-	-	-	-	-	-	-	-	-	-	Popov s.n.	KX886243*	GU004510	JQ660907
	Selenipedium aequinoctiale	-	-	-	-	-	-	-	-	-	-	-	-	826/ Szlachetko	KX886242*	GU004507	EF079360

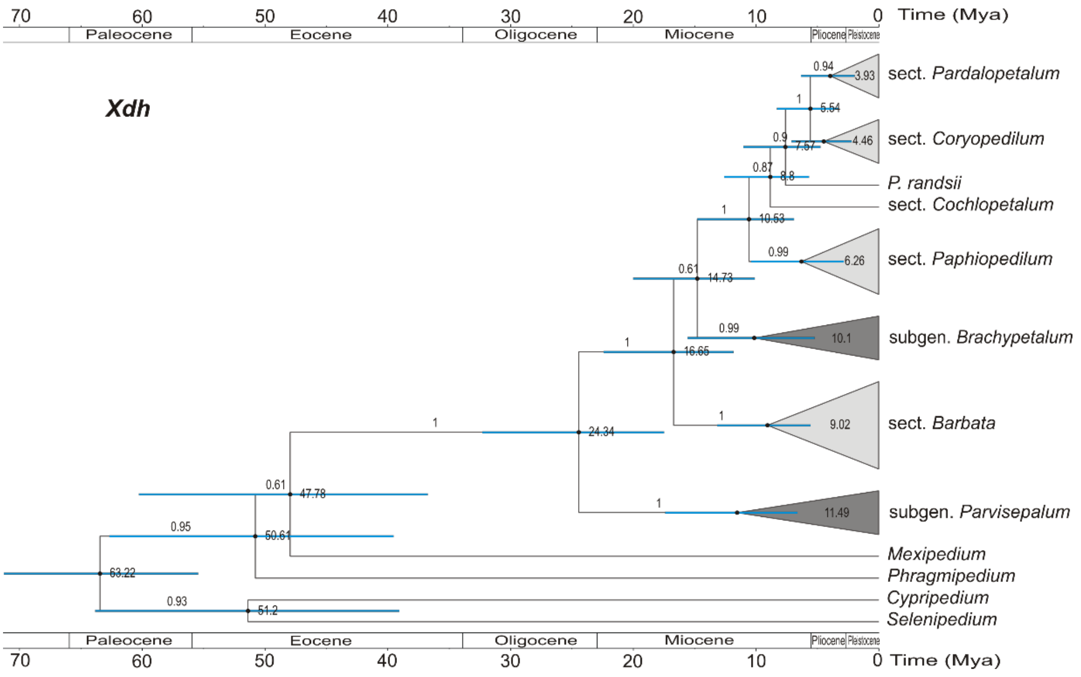

Figure S1. Time-calibrated tree of *Paphiopedilum* genus (maximum clade credibility tree) resulting from the BEAST analysis of the nuclear low copy gene *XDH*. Posterior Probability (PP) values are indicate above branches. Numbers at nodes are divergence times based on a strict clock analysis. Bars represent the 95% highest posterior credibility intervals of divergence times. Classification to clades follows Cribb (1998).

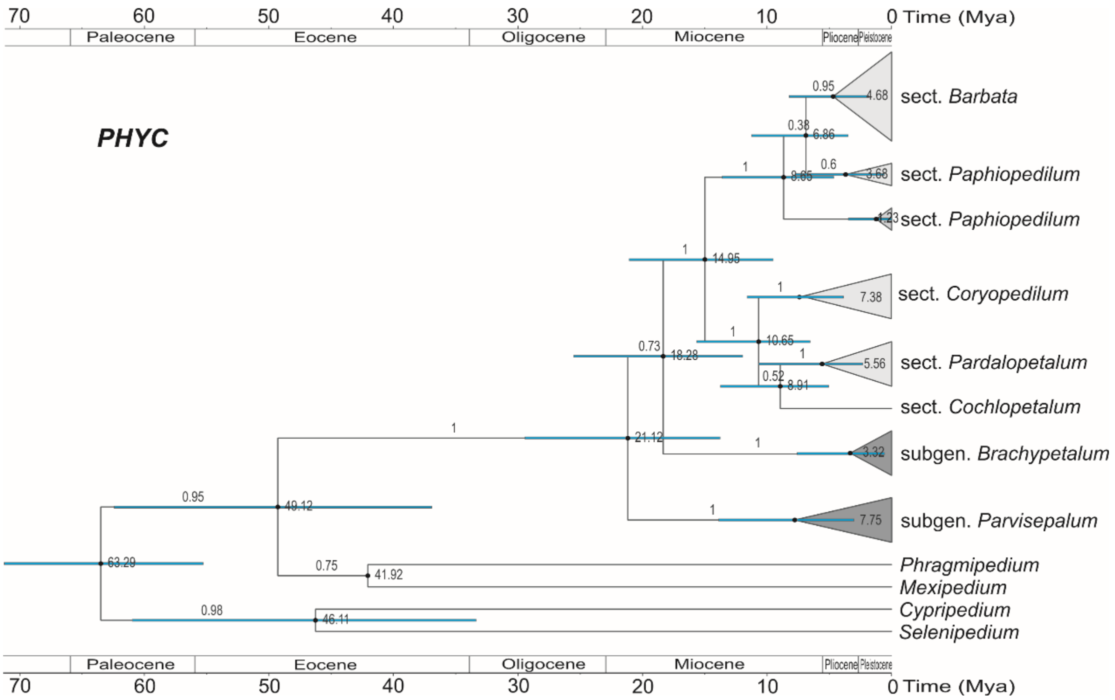

Figure S2. Time-calibrated tree of *Paphiopedilum* genus (maximum clade credibility tree) resulting from the BEAST analysis of the nuclear low copy gene *PHYC*. Posterior Probability (PP) values are indicate above branches. Numbers at nodes are divergence times based on a strict clock analysis. Bars represent the 95% highest posterior credibility intervals of divergence times. Classification to clades follows Cribb (1998).

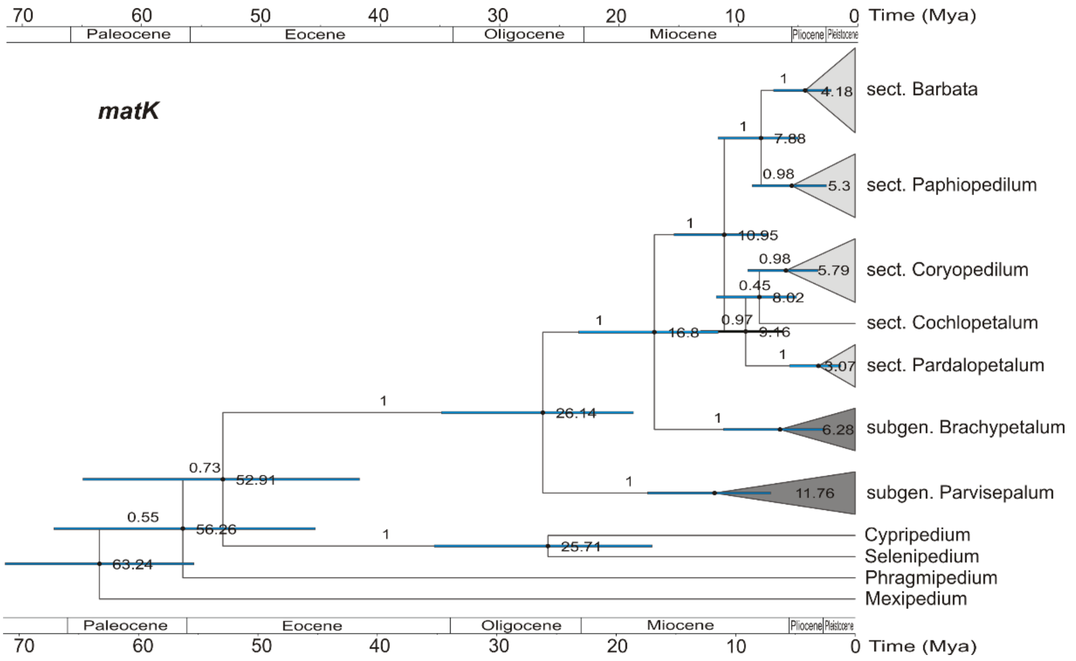

Figure S3. Time-calibrated tree of *Paphiopedilum* genus (maximum clade credibility tree) resulting from the BEAST analysis of the *matK* gene. Posterior Probability (PP) values are indicate above branches. Numbers at nodes are divergence times based on a strict clock analysis. Bars represent the 95% highest posterior credibility intervals of divergence times. Classification to clades follows Cribb (1998).

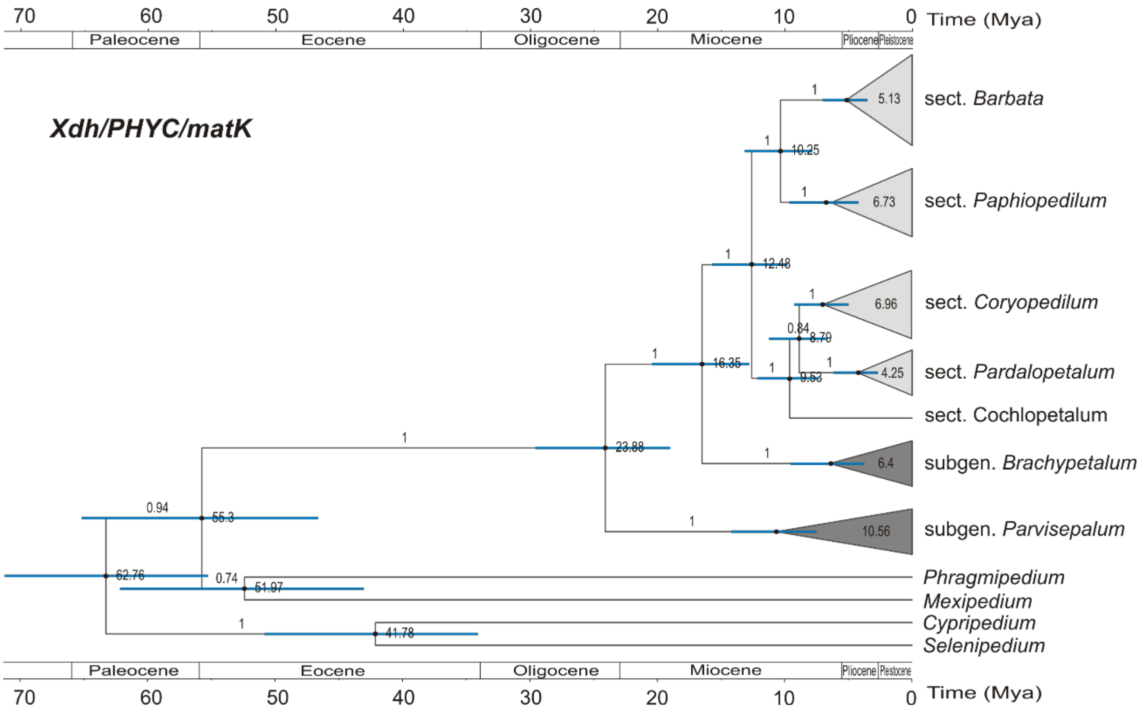

Figure S4. Time-calibrated tree of *Paphiopedilum* genus (maximum clade credibility tree) resulting from the BEAST analysis of the combined nuclear low copy gene *XDH*, *PHYC* and plastid *matK* gene. Posterior Probability (PP) values are indicate above branches. Numbers at nodes are divergence times based on a strict clock analysis. Bars represent the 95% highest posterior credibility intervals of divergence times. Classification to clades follows Cribb (1998).

References

Cribb, P.J. *The Genus Paphiopedilum.* 2nd ed.; Natural History Publications (Borneo) in association with Royal Botanic Gardens: Kew, Kota Kinabalu, Sabah, Malaysia, 1998.
